# The bone marrow endothelial progenitor cell response to septic infection

**DOI:** 10.3389/fimmu.2024.1368099

**Published:** 2024-04-04

**Authors:** Xin Shi, Kevin J. Simms, Thomas J. Ewing, Yuan-Ping Lin, Yi-Ling Chen, John N. Melvan, Robert W. Siggins, Ping Zhang

**Affiliations:** ^1^ Department of Integrative Medical Sciences, Department of Surgery, College of Medicine, Northeast Ohio Medical University, Rootstown, OH, United States; ^2^ West Clinical Laboratory, Lakeland Regional Health Medical Center, Lakeland, FL, United States; ^3^ 3R Life Sciences Ltd., Kaohsiung City, Taiwan; ^4^ Institute for Translational Research in Biomedicine, Kaohsiung Chang Gung Memorial Hospital, Kaohsiung City, Taiwan; ^5^ Memorial Cardiac and Vascular Institute, Memorial Healthcare System, Hollywood, FL, United States; ^6^ Department of Physiology, Louisiana State University Health Sciences Center, New Orleans, LA, United States

**Keywords:** endothelial progenitor cells, host defense response, bone marrow, septic infection, microvascular injury, cell signaling

## Abstract

Early increase in the level of endothelial progenitor cells (EPCs) in the systemic circulation occurs in patients with septic infection/sepsis. The significance and underlying mechanisms of this response remain unclear. This study investigated the bone marrow EPC response in adult mice with septic infection induced by intravenous injection (i.v.) of *Escherichia coli*. For *in vitro* experiments, sorted marrow stem/progenitor cells (SPCs) including lineage(lin)^-^stem cell factor receptor (c-kit)^+^stem cell antigen-1 (Sca-1)^−^, lin^−^c-kit^+^, and lin^−^ cells were cultured with or without lipopolysaccharides (LPSs) and recombinant murine vascular endothelial growth factor (VEGF) in the absence and presence of anti-Sca-1 crosslinking antibodies. In a separate set of experiments, marrow lin^−^c-kit^+^ cells from green fluorescence protein (GFP)^+^ mice, i.v. challenged with heat-inactivated *E. coli* or saline for 24 h, were subcutaneously implanted in Matrigel plugs for 5 weeks. Marrow lin^−^c-kit^+^ cells from Sca-1 knockout (KO) mice challenged with heat-inactivated *E. coli* for 24 h were cultured in the Matrigel medium for 8 weeks. The marrow pool of EPCs bearing the lin^−^c-kit^+^Sca-1^+^VEGF receptor 2 (VEGFR2)^+^ (LKS VEGFR2^+^) and LKS CD133^+^VEGFR2^+^ surface markers expanded rapidly following septic infection, which was supported by both proliferative activation and phenotypic conversion of marrow stem/progenitor cells. Increase in marrow EPCs and their reprogramming for enhancing angiogenic activity correlated with cell-marked upregulation of Sca-1 expression. Sca-1 was coupled with Ras-related C3 botulinum toxin substrate 2 (Rac2) in signaling the marrow EPC response. Septic infection caused a substantial increase in plasma levels of IFN-γ, VEGF, G-CSF, and SDF-1. The early increase in circulating EPCs was accompanied by their active homing and incorporation into pulmonary microvasculature. These results demonstrate that the marrow EPC response is a critical component of the host defense system. Sca-1 signaling plays a pivotal role in the regulation of EPC response in mice with septic infection.

## Introduction

Septic infection is a serious health problem causing enormous economic burdens and loss of life (1–6). During septic infection, invading pathogens evoke the systemic inflammatory response and cause deterioration of microcirculation with a widespread injury to endothelial cells. Structural damage and functional collapse of the microvasculature in vital organ systems is detrimental. In the lungs, for example, loss of capillary integrity and the resulting edema in terminal airways are the pathological hallmarks of adult respiratory distress syndrome (ARDS) (7), which often triggers and accelerates development of multiple organ dysfunction syndrome (MODS) with high mortality (2, 8). Therefore, compromised integrity of blood vasculature has been identified as the major cause of fatal outcomes in patients with sepsis (9). Decades of research have been devoted to studying the etiology and pathogenesis of microvascular injury as well as endothelial damage. Strikingly, host defense mechanisms for protecting the integrity of endothelium in the microvasculature remains a less explored area.

Bone marrow houses stem/progenitor cells (SPCs) including endothelial progenitor cells (EPCs). These EPCs/SPCs can be activated to participate in maintaining homeostasis of the microvasculature and repairing endothelial damage via proliferation and differentiation into endothelial cells for vascular integration as well as via production of angiogenic mediators/molecules to exert paracrine and autocrine effects on vascular endothelium (10, 11). Clinical observations have repeatedly reported that the level of EPCs in the systemic circulation increases in patients with septic infection/sepsis (12–17). This increase occurs rapidly (6–12 h) following the diagnosis of sepsis (14, 16). A greater number of circulating EPCs is associated with improved outcomes in patients with sepsis (17). A drop in EPC number in the circulation and impairment of EPC function during septic infection usually occur in association with more advanced disease, development of organ failure, and increased mortality (13, 17–20). At present, little information is available about the pattern of marrow EPC response to septic infection and the molecular signaling mechanisms underlying this response.

Since the discovery of circulating EPCs in adults by Asahara et al. in 1997 (21), constant efforts have been devoted to characterizing specific markers for EPCs. It is now commonly accepted that in humans, EPCs express lineage(lin)^−^ (or CD45^−^) CD34^+^vascular endothelial growth factor receptor 2 (VEGFR2)^+^ or CD45^−^CD34^+^CD133^+^VEGFR2^+^ surface markers (10, 11, 22). In mice, a subset of lin^−^stem cell factor receptor (c-kit)^+^stem cell antigen-1 (Sca-1)^+^ (LKS) cells expressing VEGFR2 (LKS VGFR2^+^) or expressing both CD133 and VEGFR2 (LKS CD133^+^VGFR2^+^) contain enriched EPCs (11, 23). In this study, we characterized the dynamic pattern of bone marrow EPC response and determined the role of EPCs in maintaining/restoring microvascular homeostasis in a murine model of systemic *Escherichia coli* infection. Signaling mechanisms underlying the regulation of bone marrow EPC response were also delineated.

## Materials and methods

### Animals

C57BL/6 and BALB/c mice (6–8 weeks old, both sexes) were purchased from Charles River Laboratories (Wilmington, MA) and Taconic Biosciences, Inc. (Germantown, NY). Breeding pairs of C57BL/6-Tg (UBC-GFP)30Scha/j mice were purchased from the Jackson Laboratory (Bar Harbor, ME). C57BL/6-Tg (UBC-GFP)30Scha/j mice and Sca-1^−/−^ mice (Sca-1 knockout or Sca-1 KO considered congenic on the C57BL/6 background, originally transferred from Dr. William L. Stanford’s group at the University of Toronto, Toronto, Canada) were bred under specific pathogen-free conditions in the Animal Care Facilities of Northeast Ohio Medical University, Michigan State University, and Louisiana State University Health Sciences Center. These mice were used for experiments at the age of 6–8 weeks old. All animals were housed in specific pathogen-free facilities with a 12-h light/dark cycle. Approvals from the Institutional Animal Care and Use Committees in adherence with National Institutes of Health guidelines were obtained prior to initiation of experiments.

Septic infection was induced in mice as described previously with minor modifications (24–27, 29). Briefly, an intravenous (i.v.) injection (via the jugular or penile vein) of ~1 × 10^7^ to ~1 × 10^8^ colony-forming units (CFUs) of live or heat-inactivated *Escherichia coli* (*E. coli* strain E11775 from the American Type Culture Collection, Rockville, MD) in 100 μl of pyrogen-free saline/mouse was administered. Control mice were injected with an equal volume of saline. In a subset of experiments, septic infection was induced in mice by i.v. injection of ~1 × 10^8^ CFUs of *E. coli* in 50 μl of saline/mouse. 5-Bromo-2-deoxyuridine (BrdU, 1 mg in 100 μl of phosphate-buffered saline, BD Bioscience, San Jose, CA) was i.v. administered simultaneously to each mouse. Animals were sacrificed at scheduled time points as indicated in each figure legend.

At the time of sacrifice, a heparinized blood sample was obtained by cardiac puncture. White blood cells (WBCs) were quantified under a light microscope with a hemocytometer. Plasma samples were collected after centrifugation of blood samples at 500 g for 10 min. Peripheral blood mononuclear cells (PBMCs) were isolated using lympholyte density gradient separation media 1.086 (Cedarlane, Burlington, ON, Canada). Both femurs and tibias were collected. Bone marrow cells (BMCs) were flushed out from these bones with a total volume of 2 ml of RPMI-1640 medium (Life Technologies, Grand Island, NY) containing 2% bovine serum albumin (BSA, Sigma-Aldrich, St. Louis, MO) through a 23-gauge needle. BMCs were filtered through a 70-µm nylon mesh (Sefar America Inc. Kansas City, MO). Contaminating erythrocytes in BMC samples were lysed with RBC lysis solution (Qiagen Sciences, Germantown, MD). Nucleated BMCs were washed with RPMI-1640 medium containing 2% BSA and then quantified under a light microscope with a hemocytometer.

To generate green fluorescence protein (GFP) expressing bone marrow chimeric mice, recipient wild-type C57BL/6 mice received whole-body irradiation at 900 rads/mouse and were then i.v. injected (via the jugular vein) with nucleated bone marrow cells (5 × 10^6^/mouse) from donor C57BL/6-Tg (UBC-GFP)30Scha/j mice under isoflurane anesthesia. Antibiotics (neomycin, 1.1 g/L of water, and polymyxin, 1 million U/L of water) in drinking water were given to recipient mice for 1 week before and 1 week after the bone marrow transplantation. Between 6 and 8 weeks post bone marrow transplantation, a blood sample was obtained from the tail vein of each mouse under isoflurane anesthesia to assess hematologic reconstitution by counting GFP^+^ cells under an Olympus IX81 time-lapse deconvolution fluorescent/phase-contrast microscope (Olympus America Inc. Melville, NY). In this set of experiments, GFP^+^ white blood cells (WBCs) comprised 92 ± 1% of the total WBCs in peripheral blood samples at the time of assessment. Septic infection (i.v. 1 × 10^8^ CFUs of *E. coli* in 100 µl saline/mouse) was induced in GFP bone marrow chimeric mice 12 weeks after bone marrow transplantation to collect lung samples at designated time points for morphological assessment of pulmonary recruitment of marrow cells.

In another set of experiments, C57BL/6-Tg (UBC-GFP)30Scha/j mice received i.v. challenge with either heat-inactivated (60°C for 60 min) *E. coli* (1 × 10^8^ CFUs in 100 µl of saline/mouse) or an equal volume of saline for 24 h. Marrow lin^−^ckit^+^ cells were sorted and seeded in ice-cold HC Matrigel (BD Bioscience) containing 50 ng/ml recombinant murine VEGF (BioLegend, San Diego, CA) at 1 × 10^5^ cells/ml. Matrigel (0.5 ml of Matrigel containing 50,000 cells/pouch) was implanted subcutaneously into each recipient wild-type C57BL/6 mouse for 5 weeks. Implanted Matrigel plugs were then collected to analyze vasculogenic activity in donor GFP^+^ marrow lin^−^ckit^+^ cells.

### Preparation of bacteria

For each experiment, a frozen stock culture of *E. coli* was added to tryptic soy broth and incubated for 18 h at 37°C in an orbital shaker. Bacteria were collected and washed twice with phosphate-buffered saline (PBS, Thermo Scientific, Frederic, MD). Suspension of bacteria in saline at appropriate concentrations were prepared based on its optical density at 600 nm. Actual numbers of viable bacteria were verified by standard plate counts of the bacterial suspensions on MacConkey agar plates following overnight incubation at 37°C.

### *In vitro* culture of marrow SPCs

Sorted bone marrow lin^−^ckit^+^Sca1^−^, lin^−^ckit^+^, and lin^−^ cells from naive mice were cultured in StemSpan Serum-Free Expansion Medium (StemCell Technologies, Vancouver, BC, Canada) containing 10% of mouse plasma, 100 U–100 µg/ml of penicillin–streptomycin (Thermo Fisher Scientific, Inc., Rockford, IL) with or without lipopolysaccharides (LPS, *E. coli* 0111:B4, 20–50 ng/ml, Sigma-Aldrich), and recombinant murine VEGF (50–250 ng/ml, BioLegend) in the absence and presence of anti-Sca-1 crosslinking antibodies (25 µg/ml of each clone D7 and E13, BD Biosciences) for 24 h. In one set of cultures, BrdU (10 µM, BD Biosciences) was added to the culture system during the last 4 h of culture.

### Determination of late EPC colony-forming activity in marrow SPCs

Sca-1 KO and wild-type C57BL/6 mice were i.v. challenged with heated-inactivated *E. coli* (1 × 10^8^ CFU in 100 µl of saline/mouse) or an equal volume of saline for 24 h. Marrow lin^−^ckit^+^ cells were sorted from each animal and cultured in 24-well plates. Each well contained 510 µl of Matrigel culture mixture [3 × 10^4^ cells in 510 µl of 2/3 Matrigel–1/3 endothelial progenitor outgrowth cell (EPOC) medium (BioChain Institute Inc., Newark, CA) mixture containing 25 ng of recombinant murine VEGF (BioLegend)] overlaid with 200 µl of EPOC medium containing penicillin–streptomycin (100 U–100 µg/ml). Cell cultures were maintained at 37°C in an atmosphere of 5% CO_2_. The overlaid EPOC medium was changed every 2–3 days during the cultural period of 8 weeks. Late EPC colonies formed in each well were counted under the Olympus IX81 time-lapse deconvolution fluorescent/phase-contrast microscope (Olympus America Inc.) at the end of culture.

### Flow cytometry and cell sorting

Cell phenotype, intracellular expression of specificity protein 1 (SP1), and cell BrdU incorporation were determined with flow cytometry as previously described (24–28). Briefly, nucleated BMCs and PBMCs suspended in RPMI-1640 containing 2% BSA (1 × 10^6^ cells in 100 μl medium) were added with a mixed panel of biotinylated anti-mouse lineage markers [10 µg/mL of each antibody against CD3e (clone 145-2C11), CD45R/B220 (clone RA3-6B2), CD11b (Mac-1, clone M1/70), TER-119 (clone TER-119)] and granulocyte differentiation antigen 1 (Gr-1 or Ly-6G/Ly-6C, clone RB6-8C5), or isotype control antibodies (clones A19-3, R35-95, A95-1) (BD Biosciences). Following incubation for 20 min at 4°C, flourochrome-conjugated streptavidin, anti-mouse c-kit or CD117 (clone 2B8), anti-mouse Sca-1 or Ly-6A/E (clone D7), anti-mouse VEGFR2 (CD309, Clone Avas 12α1) (BD Biosciences) anti-mouse CD133 (clone 13A4, eBiosciences, San Diego, CA), or the matched isotype control antibodies were added into the incubation system at a final concentration of 10 µg/mL for each agent. Samples were further incubated in the dark for 20 min at 4°C. Antibody-stained cells were then washed with cold PBS containing 2% BSA. For measuring cell BrdU incorporation, cells were further processed using a BD BrdU Flow Kit (BD Biosciences) with the procedure provided by the manufacturer. For measuring cell expression of cyclin D1 and SP1, cells were further processed to make both cell membrane and nuclear membrane permeable for antibody using the procedure (without the step of DNA digestion with DNase) provided by BD BrdU Flow Kit (BD Biosciences). Permeabilized cells were incubated with 10 µg/ml of fluorochrome-conjugated anti-human/mouse cyclin D1 antibody (Clone DCS-6, Thermo Fisher Scientific, Inc.) or anti-human/mouse SP1 antibody (clone E-3, Santa Cruz Biotechnology, Inc.) in the dark for 20 min at room temperature. To cell samples incubated with anti-human/mouse SP1 antibody, flourochrome-conjugated anti-mouse IgG2a, (10 µg/mL, clone R-1915, BD Biosciences) was then added. The cells were further incubated in the dark for 20 min at room temperature. The background staining control samples were incubated with the fluorochrome-conjugated second antibody only. At the end of the staining procedure, cells were washed with the washing buffer provided with the BD BrdU Flow Kit (BD Biosciences) and then suspended in 0.5 ml of PBS containing 1% paraformaldehyde. Analysis of cell phenotypes, expression of cyclin D1 and SP1, and BrdU incorporation as well as sorting of selected cell subpopulations were performed on a FACSAria Fusion flow cytometer with FACSDiva software (Becton Dickinson, San Jose, CA). Cell populations of interest were gated based on their marker or marker combinations. Depending on the cell types analyzed, the number of cells acquired in each sample was in the range of 200,000 to 300,000.

### Co-immunoprecipitation and mass spectrometry

Nucleated BMCs were isolated from Sca-1 KO and wild-type C57BL/6 mice following 24 h of systemic *E. coli* infection. Isolated BMCs were lysed with a lysing buffer (10 mM Tris-HCl buffer containing 1% Triton X-100, 5 mM EDTA, 50 mM NaCl, 30 mM sodium pyrophosphate, 2 mM sodium orthovanadate, 1 mM PMSF, 50 mM sodium fluoride, 5 mg/ml of aprotinin, 5 mg/ml of pepstatin, and 5 mg/ml of leupeptin, pH 7.6) for 30 min. After removing debris in cell lysates by centrifugation at 10,000 *g* for 5 min at 4°C, soluble protein samples in the supernatants were collected. Co-immunoprecipitation of Sca-1-associated proteins was performed using rat anti-mouse Sca-1 monoclonal antibody (clone E13-161.7, BD Biosciences) and Pierce^®^ Co-Immunoprecipitation (Co-IP) Kit (Thermo Scientific, Frederic, MD). Sca-1 co-immunoprecipitated protein fractions were resolved using the 12% SDS-PAGE ready gel (Bio-Rad Laboratories, Hercules, CA). Recovery of the specific 21-kDa band identified with Sca-1 pulldown in the SDS-PAGE gel was processed according to the typical in-gel digestion. Tryptic peptides were analyzed by Thermo Scientific Orbitrap Elite mass spectrometer coupled with Easy nano LC system (Waltham, MA). The protein matches were identified using Thermo Proteome Discoverer 1.4 with Mascot search program (matrix Science, London, UK) against the Mouse of SwissProt database. A percolator was used for the validation of peptide matches.

### Immunohistochemistry and morphological analysis

Sorted marrow lin^−^ckit^+^ cells from Sca-1 KO and wild-type C57BL/6 mice following 24 h systemic *E. coli* infection were fixed with 8% paraformaldehyde in PBS for 30 min. After washing three times with PBS containing 1% BSA, fixed cells were smeared onto poly-L-lysine-coated glass slides (Sigma-Aldrich) followed by a brief air dry. Fixed cells on slides were blocked with 10% goat serum (BioLegend) for 1 h. After cell surface staining of Sca-1 with fluorochrome-conjugated rat anti-mouse Sca-1 (clone D7, BD Biosciences) for 1 h, the cell smears were washed three times with PBS containing 1% BSA. Cells were permeabilized with 0.5% Tween-20 (Sigma-Aldrich) in PBS for 10 min. After washing three times with 0.1% Tween-20, cells on slides were sequentially re-blocked with 10% goat serum, stained with fluorochrome-conjugated rabbit polyclonal antibody against mouse Ras-related C3 botulinum toxin substrate 2 (Rac2, Bioss Inc., Woburn, MA), and eventually mounted with cover slips using the mounting medium containing 4′,6-diamidino-2-phenylindole (DAPI, Abcam, Cambridge, United Kingdom) for imaging analysis under the Olympus IX81 time-lapse deconvolution fluorescent/phase-contrast microscope (Olympus America Inc.).

Subcutaneously implanted Matrigel plug samples collected from mice were fixed with 10% paraformaldehyde in PBS for 24 h. After washing three times with PBS, the fixed samples were processed using Leica ASP300S Fully Enclosed Tissue Processor (Leica Biosystems, Deer Park, IL) and paraffin embedded with Leica Histore Arcadia H and C (Leica Biosystems). Microtome sections (5–10 µM) of paraffin-embedded tissue blocks were prepared using Leica RM2235 (Leica Biosystems). Sections were mounted on Superfrost Plus Microscope Slides (Fisher Healthcare, Pittsburgh, PA) and sequentially subjected to steps of deparaffinization, hydration, antigen retrieval, and blocking with 0.1% BSA. Slides were stained with fluorochrome-conjugated rabbit polyclone antibody against mouse von Willebrand factor (vWF, Bioss Inc.) Stained slides were mounted with cover slips using the Vectashield Antifade Mounting Medium with DAPI (Vector Laboratories, Inc. Newark, CA). Lung tissue samples collected from mice were fixed with 4% paraformaldehyde at 4°C for 24 h. After washing three times in PBS, lung tissue samples were sequentially placed into 15% sucrose solution in PBS for 2 h at 4°C and 30% sucrose solution in PBS for 24 h at 4°C. Tissue samples were then frozen with optimal cutting temperature compound (OCT; Sakura Tissue-Tek, Torrance, CA). Cryostat sections (10 µM) of OCT-embedded tissue were prepared using Leica CM1950 (Leica Biosystems). Sections were mounted on Superfrost Plus Microscope Slides (Fisher Healthcare), permeabilized with 0.2% Triton (Trition X-100, Sigma-Aldrich), and blocked with Normal Serum Block (Biolegend). Slides were stained with fluorchrome-conjugated Lycopresision Esculentum Lectin (isolectin B4, Thermo Fisher Scientific Inc.). Stained slides were mounted with cover slips using Vectashield Antifade Mounting Medium with DAPI (Vector Laboratories, Inc.) for imaging analysis under an Olympus FV1000 IX81 Spectral Confocal Microscope (Olympus America Inc.).

### Real-time RT-PCR

Total RNA samples were prepared from marrow lin^−^c-kit^+^Sca-1^−^ and lin^−^c-kit^+^ cells with RNeasy Plus Mini Kit (Qiagen, Valencia, CA). Real-time RT-PCR analysis of mRNA expression by cells was performed as reported previously (24). Each RNA sample was subjected to two-step real-time RT-PCR using iScriptTM Reverse Transcription Supermix kit and SsoFastTM EvaGreen^®^ Supermix kit (Bio-Rad Laboratories), respectively, on a CFX96TM Real-Time System (Bio-Rad Laboratories). The amplification primer pairs were as follows:

Sca-1: Forward 5′-GTTTGCTGATTCTTCTTGTGGCCC

Reverse 5′-ACTGCTGCCTCCTGAGTAACAC

VEGFR2: Forward 5′-ATCCAGATGAGGGCAAGTTTAG

Reverse 5′-GCATACCCACTGACTGTGATAG

18SrRNA: Forward 5′-ATTCGAACGTCTGCCCTATAA

Reverse 5′-GTCACCCGTGGTCACCATG

These sets of primers were designed using Primer Express software (Life Technologies Co, Carlsbad, CA). The expression of Sca-1 and VEGFR2 mRNA was determined by normalizing the cycle threshold (CT) number of their individual mRNA with that of 18S rRNA in each sample. Changes in specific gene mRNA expression by cells from groups with different treatments are expressed as fold alterations over the baseline expression by cells from the corresponding control group.

### ELISA determination

Lung tissue homogenate (10%) was prepared by homogenizing lung tissue in PBS containing cOmplete Tablets Mini protease inhibitor cocktail (1 tablet/10 ml of PBS, Sigma-Aldrich) with a TH-115 homogenizer (OMNI International, Kennesaw GA). After centrifugation at 10,000 *g* for 10 min, the supernatant of each tissue homogenate sample was collated. VEGFR2, SDF-1, and IFN-γ concentrations in plasma and lung tissue homogenate samples were determined with Quantikine Mouse VEGF Immunoassay kit, Mouse CXCL12/SDF-1 Immunoassay Kit, and Mouse IFN-γ Immunoassay Kit (R&D Systems, Minneapolis, MN), respectively. G-CSF level in plasma and lung tissue homogenate samples were determined with the Mouse CSF3 (G-CSF) ELISA Kit (Thermo Scientific). Protein content in lung tissue homogenate samples was determined using the BCA Protein Assay Kit (Thermo Fisher Scientific, Inc.) with the protocol provided by the manufacturer.

### Statistical analysis

Data were presented as mean ± SEM. The sample size is indicated in each figure legend. Statistical analysis was conducted using Student’s *t*-test, one-way ANOVA followed by Student–Newman–Keuls test, liner correlation, and Chi-square test. Difference with statistical significance is accepted at p < 0.05.

## Results

### Change in EPC subpopulations in the bone marrow following septic infection

**Figure 1:**
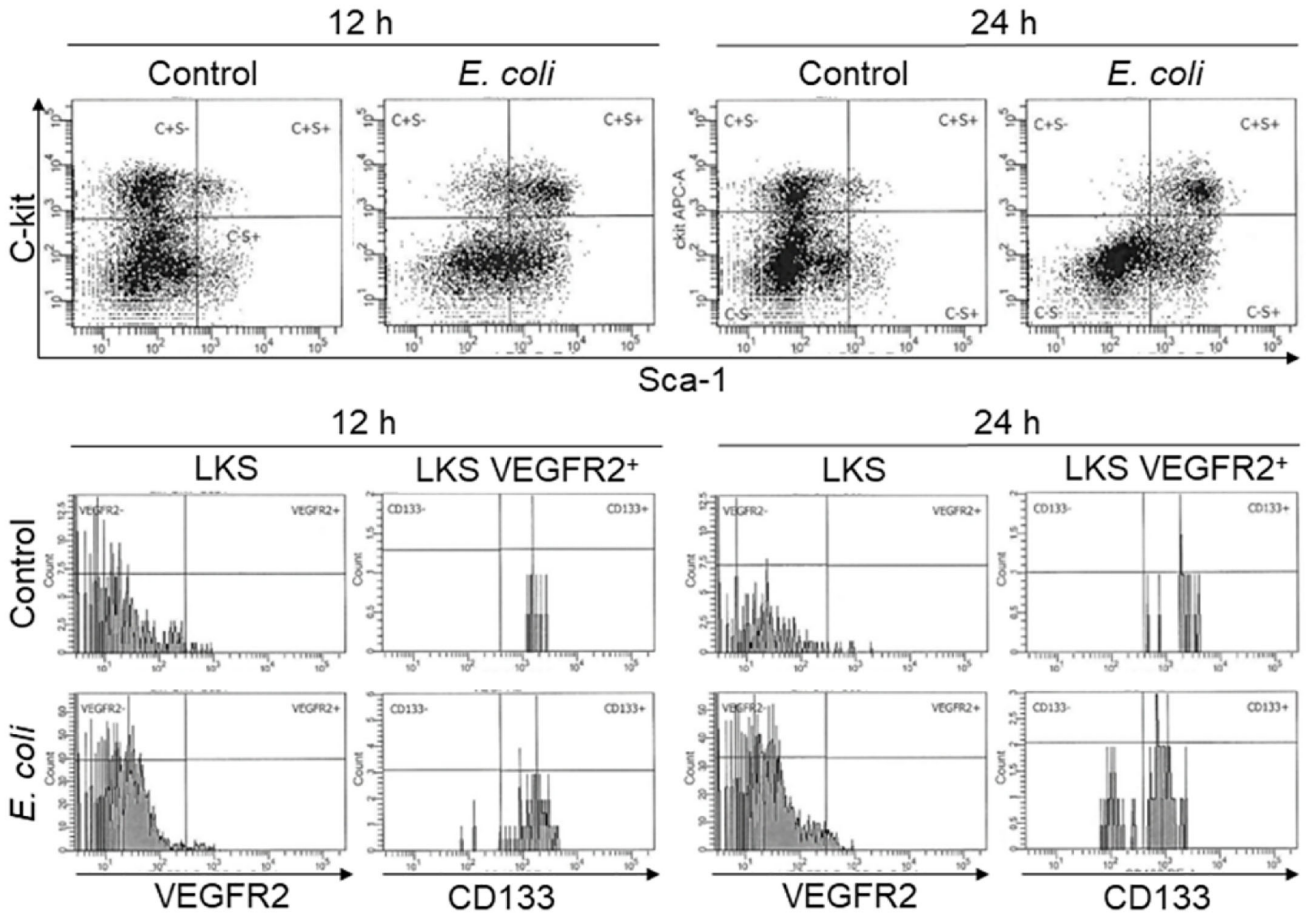
Representative plots of flow cytometry for analyzing changes in bone marrow LKS, LKS VEGFR2^+^, and LKS CD133^+^VEGFR2^+^ cell subtypes in mice 12 and 24 h following i.v. challenge with saline (control) or *E. coli* (1 × 10^8^ CFUs/mouse).

**Figure 2:**
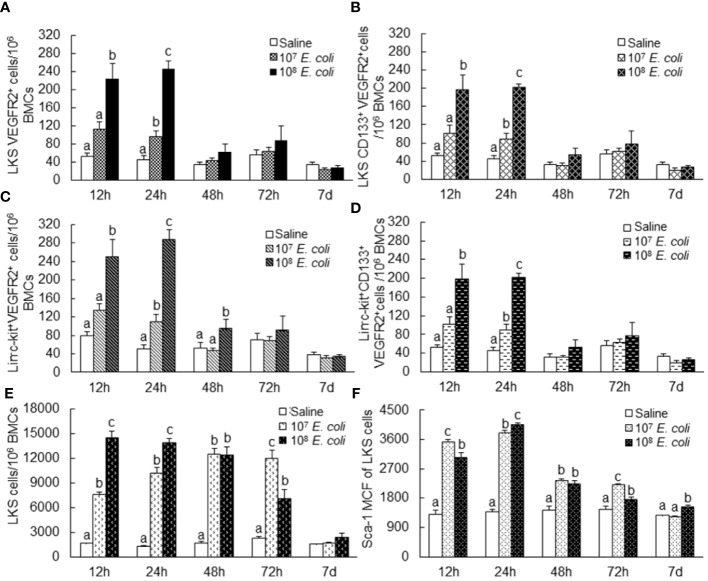
Changes in bone marrow LKS VEGFR2^+^ **(A)**, LKS CD133^+^ VEGFR2^+^ **(B)**, lin^−^c-kit^+^VEGFR2^+^ **(C)**, lin^−^c-kit^+^CD133^+^VEGFR2^+^ **(D)**, and LKS **(E)** cell populations as well as Sca-1 expression [as reflected by mean channel fluorescence intensity (MCF)] by LKS cells **(F)** following septic infection. N = 5–10. Data are mean ± SEM. Bars with different letters at each time point in each panel are statistically different (p < 0.05).

**Figure 3:**
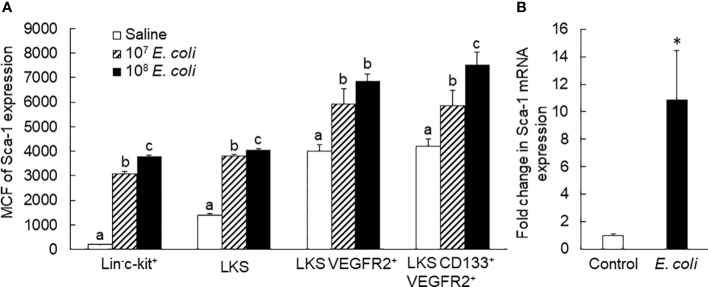
**(A)** Sca-1 protein expression by bone marrow stem/progenitor cell types 24 h following bacteremia. MCF, mean channel fluorescence intensity. N = 5. Data are mean ± SEM. Bars with different letters in each cell type are statistically different (p < 0.05). **(B)** Fold change in Sca-1 mRNA expression by bone marrow lin^−^c-kit^+^ cells 24 h following i.v. challenge with *E coli* (~1 × 10^8^ CFUs/mouse). N = 4. Data are mean ± SEM. *p < 0.05.

To characterize the pattern of marrow EPC response, we analyzed the dynamic alternations of EPC subsets in nucleated BMCs in mice following systemic *E. coli* infection. **Figure 1** exhibits representative dot plots and histograms of flow cytometry for determining marrow LKS, LKS VGFR2^+^, and LKS CD133^+^VGFR2^+^ cell subsets at 12 and 24 h in mice challenged with i.v. *E. coli* (1 × 10^8^ CFUs/mouse) vs. controls receiving i.v. saline. **Figure 2** summarizes changes in LKS VEGFR2^+^ (A), LKS CD133^+^ VEGFR2^+^ (B), lin^−^c-kit^+^VEGFR2^+^ (C), lin^−^c-kit^+^CD133^+^VEGFR2^+^(D), and LKS (E) cell populations, as well as Sca-1 expression [as reflected by mean channel fluorescence intensity (MCF)] by LKS cells (F) for 7 days following septic infection. EPC subpopulations in nucleated BMCs rapidly expanded following systemic *E. coli* infection. At 12 h post i.v. *E. coli* at 1 × 10^7^ CFUs/mouse, the numbers of both marrow LKS VGFR2^+^ and LKS CD133^+^ VGFR2^+^ EPCs tended to increase in comparison to those of controls, although these increases did not reach statistical significance. Mice challenged with *E. coli* at 1 × 10^8^ CFUs/mouse showed marked increases in both LKS VGFR2^+^ and LKS CD133^+^ VGFR2^+^ EPCs in the bone marrow in comparison to those of the control group and 1 × 10^7^ CFU *E. coli* group (p < 0.05). At 24 h post i.v. *E. coli* at 1 × 10^7^ CFUs/mouse, the numbers of both marrow LKS VGFR2^+^ and LKS CD133^+^ VGFR2^+^ phenotypes of EPCs increased significantly compared to those of the control groups (p < 0.05). Mice challenged with *E. coli* at 1 × 10^8^ CFUs/mouse showed further increases in both marrow LKS VGFR2^+^ and LKS CD133^+^ VGFR2^+^ EPCs than did the 1 × 10^7^ CFU *E. coli* group (p < 0.05). After 48 h of *E. coli* infection, the marrow EPC pools returned to the control levels in both the 1 × 10^7^ and 1 × 10^8^ CFUs/mouse groups. These results indicate that the marrow EPC response occurs in the early stage of septic infection. The EPC response intensifies with the increase in the severity of bacterial infection. Our previous studies have demonstrated that both activation of LKS cell proliferation and phenotypic conversion to LKS-type cells from the downstream lin^−^c-kit^+^Sca-1^−^ cells via their reexpression of Sca-1 contribute to the expansion of marrow LKS cell pool during septic infection (29). Since EPCs are subpopulations of LKS cells, we simultaneously analyzed the net change in numbers of VGFR2^+^ and CD133^+^VGFR2^+^ cells in marrow lin^−^c-kit^+^ cell population, which contained both lin^−^c-kit^+^Sca-1^−^ and lin^−^c-kit^+^Sca-1^+^ cells. The results showed that marrow lin^-^c-kit^+^VGFR2^+^ and lin^−^c-kit^+^CD133^+^VGFR2^+^ cells increased substantially at 12 and 24 h post i.v. *E. coli*. This net increase in VGFR2^+^ and CD133^+^VGFR2^+^ cells in marrow lin^−^c-kit^+^ cell pool suggested the contribution of cell proliferation to the increase in the entire cell pool. In agreement with our previous studies, septic infection caused a substantial expansion of marrow LKS cell population. However, the pattern of increase in marrow LKS cells was not the same as those in marrow EPC subtypes. Specifically, the increase in marrow EPCs occurred in the early phase (12 and 24 h) of septic infection, Whereas the increase in the general LKS cell population in bone marrow lasted to 72 h following i.v. *E. coli*. To understand why the continued increase in marrow LKS cells during 48–72 h following septic infection did not persistently support the increase in marrow LKS VGFR2^+^ and LKS CD133^+^VGFR2^+^ EPCs, we analyzed the level of Sca-1 expression by these cells. The MCF level of Sca-1 expression by LKS VGFR2^+^ and LKS VGFR2^+^CD133^+^ EPCs was above 3,000 in all groups (**Figure 3A**), Whereas marrow LKS cells exhibited elevation of Sca-1 MCF above this level only in the 12- and 24-h groups of septic infection (**Figure 2F**). Septic infection induced upregulation of Sca-1 expression by marrow SPCs in the lin^−^c-kit^+^ cell population that was involved in the activation of Sca-1 gene transcription (**Figure 3B**). These observations suggested that the marked upregulation of Sca-1 expression might play a critical role in the marrow EPC response.

### Activation of proliferative and vasculogenic activities in marrow EPCs following septic infection

**Figure 4:**
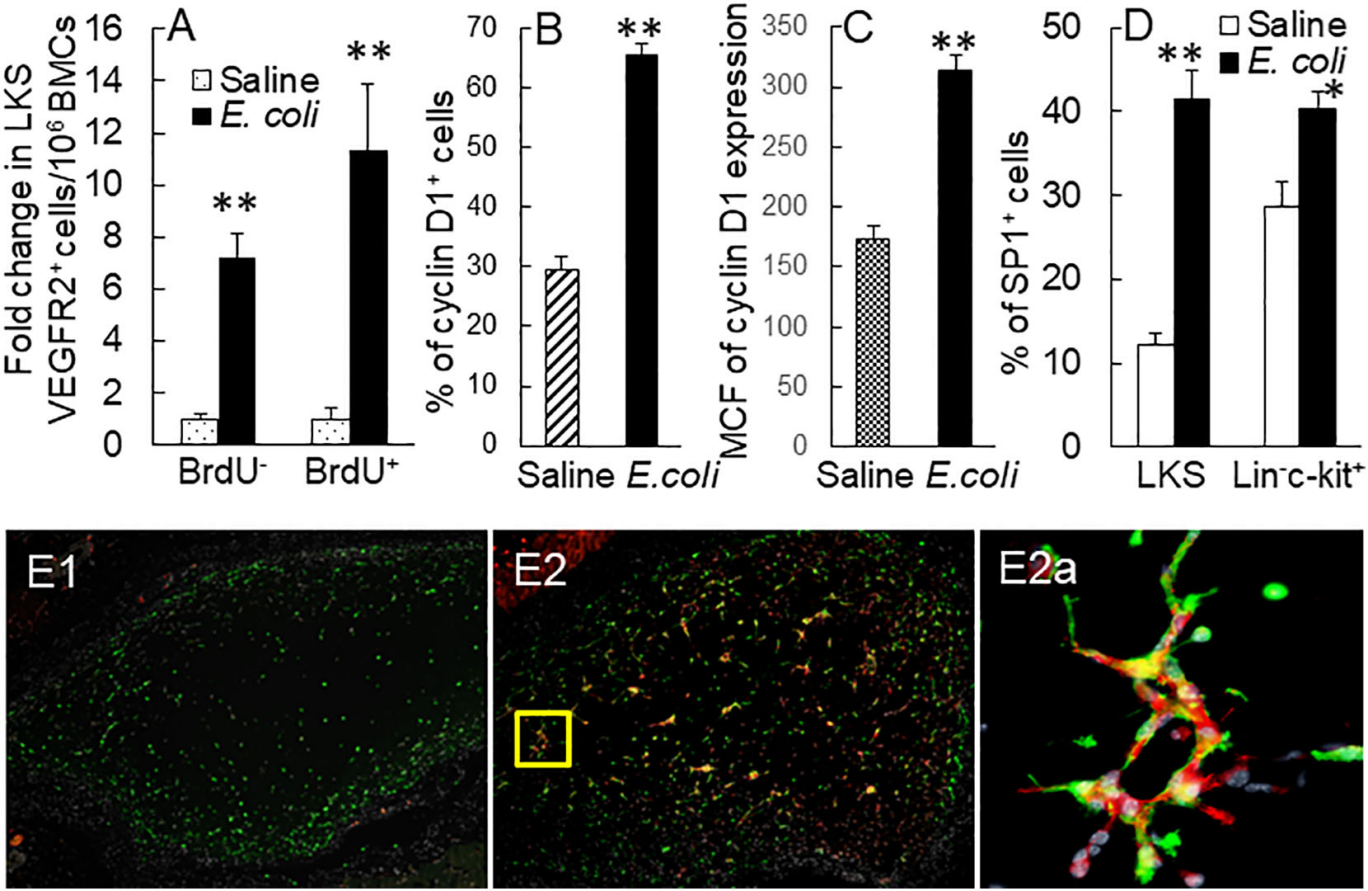
**(A)** Fold changes in bone marrow BrdU^+^ and BrdU^−^ LKS VEGFR2^+^ cells in mice 24 h following i.v. challenge with *E. coli* (~1 × 10^8^ CFUs/mouse). N = 5. Data are mean ± SEM. **p < 0.01 vs. the correspondent saline group. **(B, C)** Upregulation of cyclin D1 expression by marrow lin^−^c-kit^+^ cells 24 h following i.v. challenge with *E. coli* (~5 × 10^7^ CFUs/mouse). Data are mean ± SEM. MCF, mean channel fluorescence intensity. N = 5. **p < 0.01 vs. the saline group. **(D)** Increase in SP1^+^ cells in bone marrow LKS and lin^−^c-kit^+^ cell subpopulations 18 h post i.v. challenge with *E. coli* (~5 × 10^7^ CFUs/mouse). N = 5. Data are mean ± SEM. *p < 0.05 compared to the corresponding saline group; **p < 0.01 compared to the corresponding saline group. **(E1, E2)** Representative images of 5-week vasculogenic activity in implanted Matrigel plugs containing bone marrow lin^−^c-kit^+^ cells from donor GFP^+^ mice challenged with i.v. saline and heat-inactivated *E. coli* (~1 × 10^8^ CFUs/mouse), respectively, for 24 h. **(E2a)** An amplified image in the area framed by the yellow square in **(E2)**.

*In vivo* BrdU incorporation analysis exhibited that proliferation of marrow EPCs (BrdU^+^ LKS VGFR2^+^) was markedly enhanced 24 h post i.v. *E. coli* (**Figure 4A**). This increase in cell proliferation contributed to the expansion of marrow EPC pool. Our previous investigations have demonstrated that systemic *E. coli* infection in mice activates the TLR4-extracellular signal-regulated kinases 1 and 2 (ERK1/2) pathway in marrow SPCs (24, 26). TLR4-ERK1/2 signaling mediates marrow SPC proliferation via upregulation of cyclin D1/cyclin-dependent kinases 4 and 6 (CDK4/6) activity. Septic infection caused a significant upregulation of cyclin D1 expression by marrow cells (**Figures 4B, C**). Concurrently, the number of BrdU^−^ LKS VGFR2^+^ cells also significantly increased in the bone marrow. This increase in BrdU^−^ LKS VGFR2^+^ cells mainly resulted from phenotypic conversion of lin^−^c-kit^+^Sca-1^−^ cells to LKS cells via reexpression of Sca-1. TLR4-ERK1/2 signaling mediates SP1 expression (30, 31). The promoter of VEGFR2 gene contains SP1-binding sites and responds to SP1 (32). A substantial increase in SP1 expression was observed in marrow lin^−^c-kit^+^, particularly by LKS cells 18 h following i.v. challenge with *E. coli* (**Figure 4D**). The activation of TLR4-ERK1/2-SP1 signaling might mediate VEGFR2 expression by marrow SPCs. As described above, the number of VGFR2^+^ cells in marrow lin^−^c-kit^+^ cell pool increased substantially at 12 and 24 h following septic infection (**Figure 2C**). Except for activation of cell proliferation, upregulation of VEGFR2 expression by cells might potentially contribute to this rapid increase in VGFR2^+^ cells in marrow lin^−^c-kit^+^ cell pool. To determine the alteration in vasculogenic activity in marrow SPCs, marrow lin^−^c-kit^+^ cells from donor GFP^+^ mice challenged with i.v. saline and heat-inactivated *E. coli* (~1 × 10^8^ CFUs/mouse) for 24 h were seeded in Matrigel and then implanted subcutaneously in wild-type C57BL/6 recipient mice. As shown in **Figure 4E1**, GFP^+^ cells from doner mice that received i.v. saline were present in Matrigel plugs without active vasculogenesis [mostly negative for PE-conjugated anti-mouse vWF staining (endothelial cell marker in red fluorescence), zero of five plugs] 5 weeks following subcutaneous implantation, whereas two of three Matrigel plugs containing GFP^+^ cells from doner mice challenged with i.v. heat-inactivated *E. coli* showed markedly enhanced activity of vasculogenesis (positive for vWF expression stained in red fluorescence, **Figure 4E2**). In the amplified image of a typical region selected from **Figure 4E2**, the characteristic vasculogenesis in forming microvascular islands was clearly visualized (**Figure 4E2a**). These results demonstrate the expansion of marrow EPC pool occurring along with activation of EPC proliferation and enhancement of EPC vasculogenic activity in response to septic infection.

### Enhancement of Sca-1 expression correlated with expansion of the EPC subpopulation in marrow SPCs

**Figure 5:**
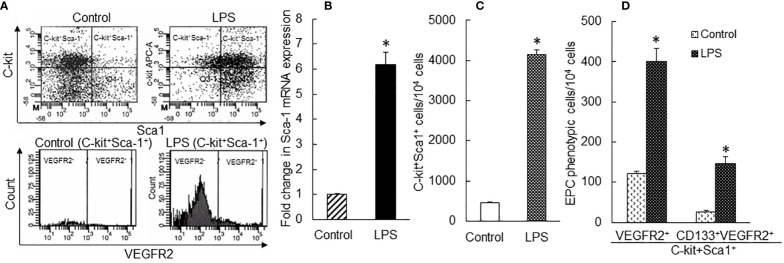
**(A)** Representative flow cytometry plots of phenotypic conversion to c-kit^+^Sca-1^+^ cells and increase in VEGFR2-expressing c-kit^+^Sca-1^+^ cells in cultured marrow lin^−^c-kit^+^Sca-1^−^ 24 h following LPS stimulation. **(B)** Upregulation of Sca-1 mRNA expression by cultured bone marrow lin^−^c-kit^+^Sca-1^−^ cells 24 h following LPS stimulation. N = 3. *p < 0.01 vs. the control group. **(C, D)** Changes in cell subpopulations in cultured marrow lin^−^c-kit^+^Sca-1^−^ 24 h following LPS stimulation. N = 5. Data are means ± SEM. *p < 0.01 vs. the correspondent control group of the same cell subpopulation.

**Figure 6:**
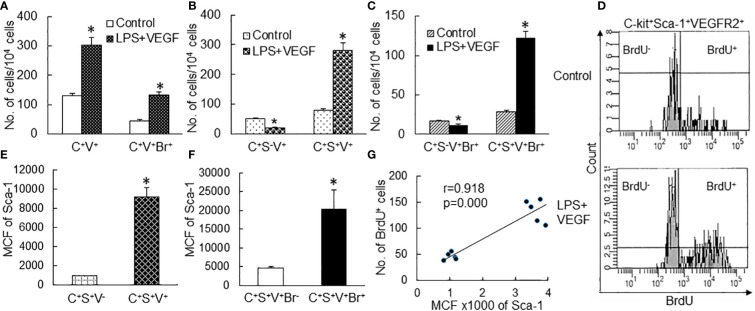
**(A–C)** Changes in cell subtypes in cultured bone marrow lin^−^c-kit^+^Sca-1^−^ cells 24 h following LPS + VEGF stimulation. N = 5. Data are means ± SEM. *p < 0.01 vs. the corresponding control group of the same cell subtype. **(D)** Representative flow cytometry plots of increase in BrdU incorporation into c-kit^+^Sca-1^+^VEGFR2^+^ cell subtype in cultured bone marrow lin^−^c-kit^+^Sca-1^−^ cells 24 h following LPS + VEGF stimulation. **(E, F)** Sca-1 expression by different cell subtypes in bone marrow lin^−^ cells cultured for 24 h in the absence and presence of LPS + VEGF stimulation. MCF, mean channel fluorescence intensity. N = 10. Data are means ± SEM. *p < 0.01 vs. the corresponding control group. **(G)** The significant correlation between Sca-1 expression and BrdU incorporation in bone marrow lin^−^c-kit^+^Sca-1^−^ cells cultured for 24 h. C^+^V^+^, c-kit^+^VEGFR2^+^; C^+^V^+^Br^+^, c-kit^+^VEGFR2^+^BrdU^+^; C^+^S^−^V^+^, c-kit^+^Sca-1^−^VEGFR2^+^; C^+^S^+^V^+^, c-kit^+^Sca-1^+^VEGFR2^+^; C^+^S^−^V^+^Br^+^, c-kit^+^Sca-1^-^VEGFR2^+^BrdU^+^; C^+^S^+^V^+^Br^+^, c-kit^+^Sca-1^+^VEGFR2^+^BrdU^+^; C^+^S^+^V^−^, c-kit^+^Sca-1^+^VEGFR2^−^; C^+^S^+^V^+^Br^−^, c-kit^+^Sca-1^+^VEGFR2^+^BrdU^−^.

To further define the correlation between upregulation of Sca-1 expression and expansion of the EPC subpopulation in marrow SPCs, we conducted a set of *in vitro* studies in which freshly sorted marrow lin^−^c-kit^+^Sca-1^−^ cells from naïve mice were cultured for 24 h with or without LPS stimulation. **Figure 5A** shows the representative plots of flow cytometry exhibiting the reexpression of Sca-1 expression by cultured lin^−^c-kit^+^Sca-1^−^ cells and increase in VEGFR2 expression by converted c-kit^+^Sca-1^+^ cells following LPS stimulation. LPS-stimulated reexpression of Sca-1 by cultured lin^−^c-kit^+^Sca-1^−^ cells was supported by a marked increase in Sca-1 gene transcription (**Figure 5B**). Concomitantly, numbers of converted c-kit^+^Sca-1^+^ cells as well as EPC subtype cells with c-kit^+^Sca-1^+^VGFR2^+^ and c-kit^+^Sca-1^+^CD133^+^VGFR2^+^ surface markers were substantially increased in the culture system with LPS stimulation (**Figures 5C, D**). In another set of *in vitro* study on cell BrdU incorporation, freshly sorted marrow lin^−^c-kit^+^Sca-1^−^ cells were cultured for 24 h in the absence and presence of LPS plus recombinant murine VEGF. LPS plus VEGF stimulation significantly increased VEGFR2^+^ cells in total c-kit^+^ cell population and BrdU incorporation into c-kit^+^VEGFR2^+^ cells (**Figure 6A**). LPS plus VEGF treatment reduced the number of c-kit^+^Sca-1^-^VEGFR2^+^ cells and BrdU incorporation into these cells in the culture system (**Figures 6B, C**). In contrast, LPS plus VEGF treatment markedly increased the number of c-kit^+^Sca-1^+^VEGFR2^+^ cells and BrdU incorporation into c-kit^+^Sca-1^+^VEGFR2^+^ cells in the culture system. **Figure 6D** shows the representative histograms of flow cytometry determining the increase in BrdU incorporation into c-kit^+^Sca-1^+^VEGFR2^+^ cells in response to LPS plus VEGF treatment. C-kit^+^Sca-1^+^VEGFR2^+^ cells expressed a much higher level of Sca-1 in comparison to c-kit^+^Sca-1^+^VEGFR2^-^ cells (**Figure 6E**). Furthermore, proliferative (BrdU^+^) c-kit^+^Sca-1^+^VEGFR2^+^ cells expressed a substantially higher level of Sca-1 than did non-proliferative (BrdU^−^) c-kit^+^Sca-1^+^VEGFR2^+^ cells (**Figure 6F**). Correlation analysis demonstrated that the proliferative activity (BrdU incorporation) was highly correlated with Sca-1 expression in the cultured marrow lin^−^c-kit^+^Sca-1^−^ cells (**Figure 6G**).

### Alteration in angiogenic factor production in the plasma and lung tissue

**Figure 7:**
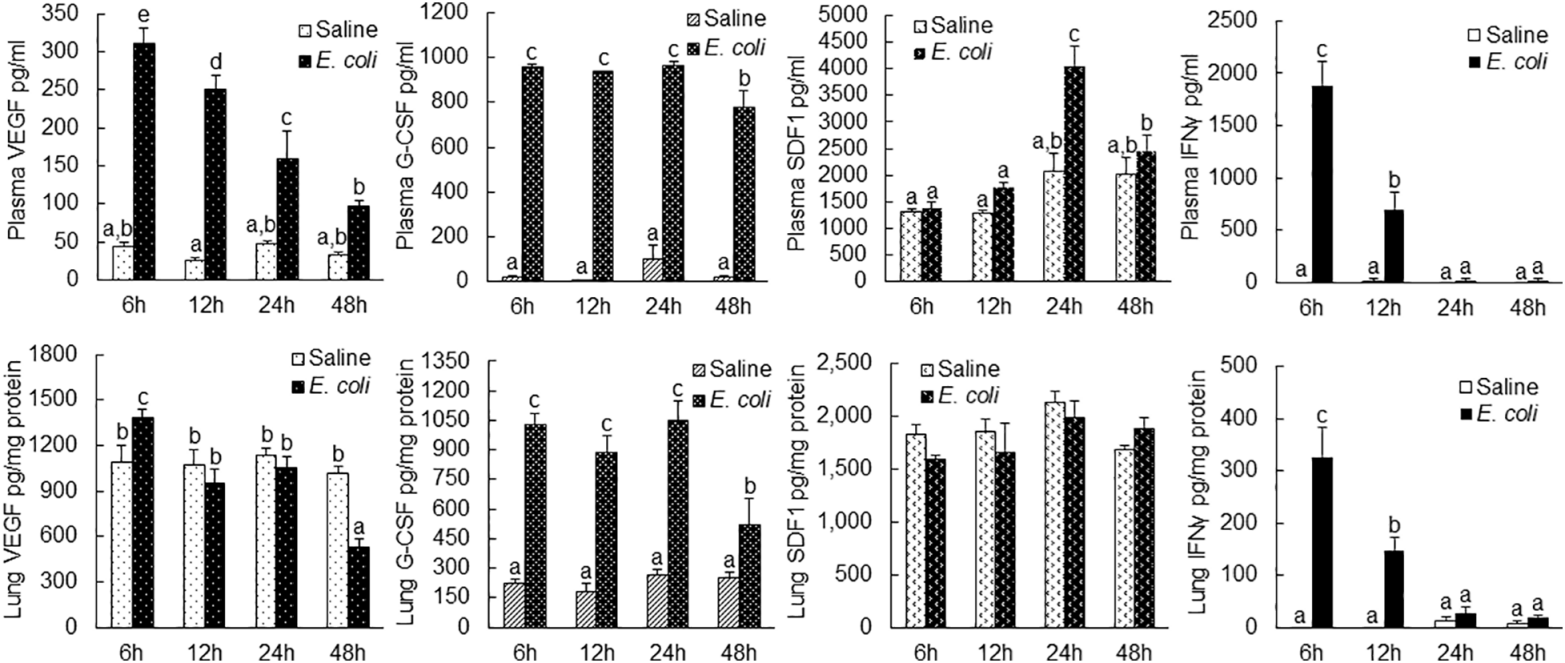
Changes in plasma and lung tissue cytokine levels following i.v. challenge with *E. coli* (~1 × 10^8^ CFUs/mouse). N = 5. Data are mean ± SEM. Bars with different letters in each panel are statistically different (p < 0.05).

VEGF level in the plasma increased substantially at 6 h post i.v. *E. coli* (**Figure 7**). The substantial elevation of plasma VEGF level gradually reduced after the 6-h time point but remained significantly higher in comparison to the control level at 24 h of septic infection. In lung tissue, VEGF level was moderately increased (with statistical significance) only at 6 h post i.v. *E. coli*. At 48 h of septic infection, the VEGF level in lung tissue was significantly lower than that in controls. G-CSF levels were markedly increased in both plasma and lung tissue between 6 and 48 h following septic infection. The increase in SDF-1 level was only seen in the plasma at 24 h post i.v. *E. coli*. In addition, IFN-γ levels in both plasma and lung tissue were substantially elevated at 6 and 12 h following septic infection.

### Increase in circulating EPC level and recruitment of marrow-derived EPCs into the lung microvasculature

**Figure 8:**
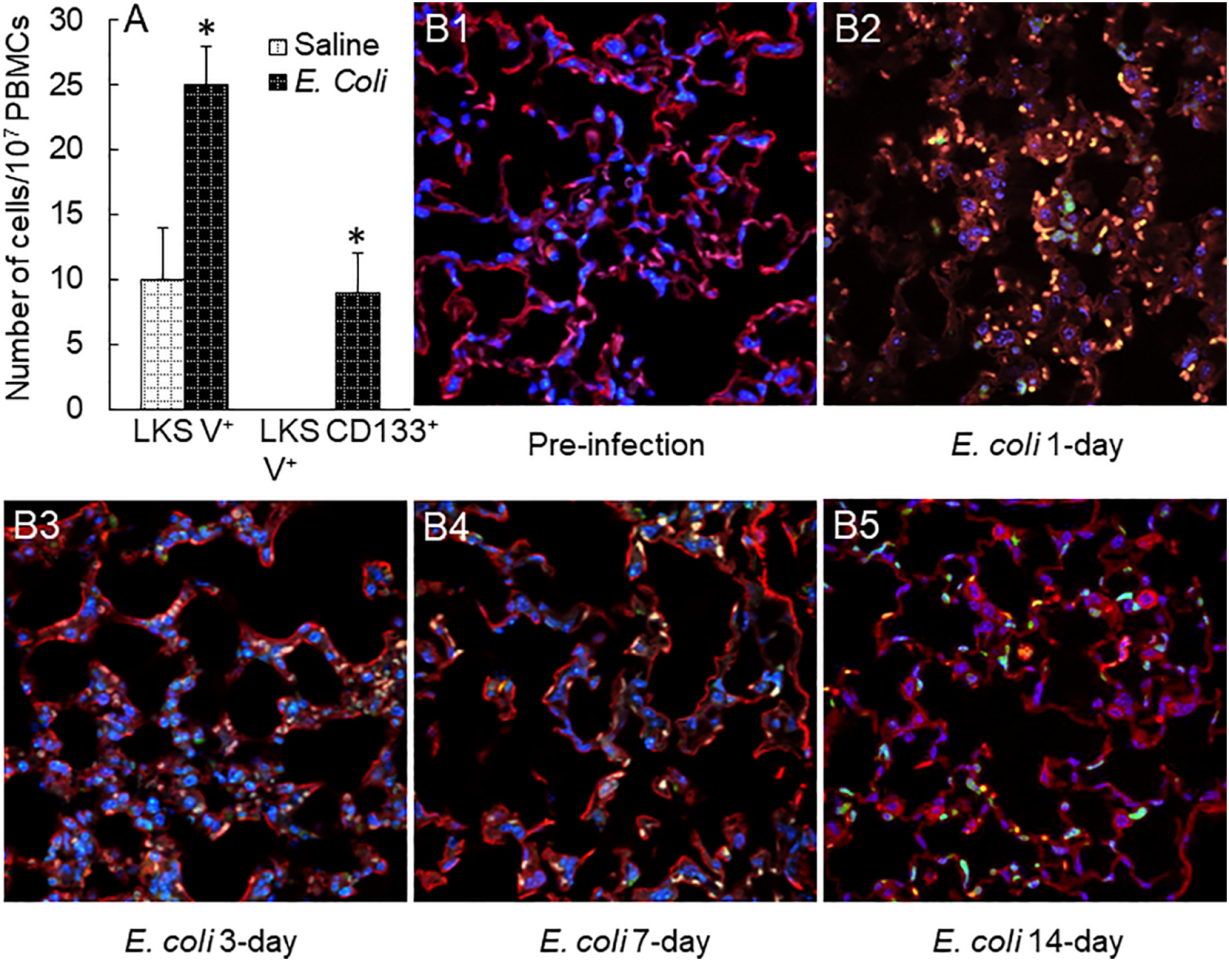
**(A)** Increase in circulating EPCs in mice 48 h following i.v. challenge with *E. coli* (~1 × 10^8^ CFUs/mouse). N = 5. Data are mean ± SEM. *p < 0.05 vs. saline group. V^+^, VEGFR2^+^. **(B1–5)** Changes in bone marrow cell recruitment into the lungs in mice challenged with i.v. *E. coli* (~1 × 10^8^ CFUs/mouse). Images are representatives of four to seven experiments.

With the rapid expansion of marrow EPC pool, the levels of LKS VEGFR2^+^ and LKS CD133^+^VEGFR2^+^ EPCs in the systemic circulation markedly increased 48 h post i.v. *E. coli* (**Figure 8A**). Since EPCs in the systemic circulation generally represent their temporary status of traveling, we employed a chimeric mouse model with reconstituted GFP^+^ bone marrow to monitor the destination of marrow-released EPCs during host response to septic infection. As shown in **Figures 8B1–5**, prior to septic infection, normal lung tissue contained very few (if not none) cells derived from the bone marrow with positive expression of GFP. At 24 h following septic infection, massive GFP^+^ cells (in green fluorescence) were recruited into the lungs, indicating that the bone marrow served as a major source of cells for cell infiltration into tissue sites of inflammation. In the meantime, the alveolar walls were markedly widened reflecting the development of tissue edema. The number of GFP^+^ cells was apparently reduced in the lungs by 72 h of septic infection. Nevertheless, many GFP^+^ cells remained in the lumen of the microvasculature. Some of these recruited cells attached/integrated to the capillary wall as shown by colocalization of GFP^+^ cells with endothelial cells stained positively by PE-labeled isolectin B4 (red fluorescence). In addition, some GFP^+^ cells migrated into the alveolar spaces. Interstitial edema in the alveoli appeared to start subsidence. These dynamic changes in morphology within 72 h of septic infection represented the characteristic feature of acute inflammation in the terminal airways. According to the classical concept of the inflammatory response, most of these recruited cells at this stage were marrow-derived phagocytes (granulocytes and monocytes) for enhancing local host defense. By day 7 post systemic *E. coli* infection, however, most of the marrow-derived inflammatory cells, previously sequestered in the microvasculature, disappeared leaving many GFP^+^ cells integrated into the capillary wall (colocalization of green and red fluorescence), which suggests that circulating EPCs released from the expanded marrow EPC pool are actively homing to the lungs and participating in maintaining and/or restoring the integrity of the microvasculature following septic infection. On day 14 post septic infection, the morphology of the lung tissue returned to normal appearance leaving the integration of many GFP^+^ cells in the capillary wall in alveoli.

### Activation of Sca-1 signaling in the marrow EPC response

**Figure 9:**
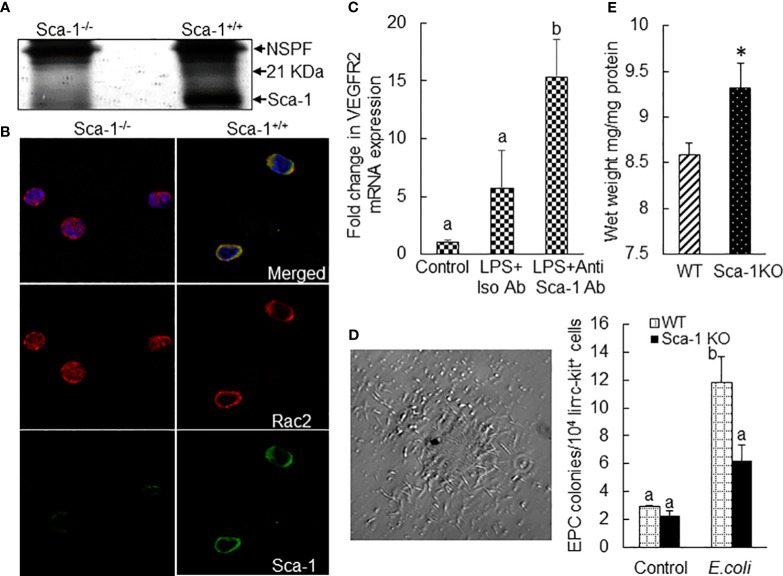
**(A)** SDS-PAGE (silver stain) exhibition of a 21-kDa protein fraction co-immunoprecipitated with Sca-1 in nucleated bone marrow cell lysates from mice 24 h following systemic *E coli* infection (i.v. ~1 × 10^7^ CFUs/mouse). NSPP, non-specific pulldown fraction. N = 3. Mass spectrometry identified enrichment of Rac2 in this 21-kDa protein fraction. **(B)** Co-localized surface expression of Sca-1 and Rac2 by marrow precursor cells in mice 24 h following system *E coli* infection (i.v. ~1 × 10^7^ CFUs/mouse). The images of confocal microscopy are representatives of three experiments. **(C)** Fold changes in VEGFR2 mRNA expression by mouse bone marrow lin^−^ckit^+^ cells cultured for 24 h in the absence and presence of LPS plus Sca-1 crosslinking antibodies. N = 5. Data are mean ± SEM. Bars with different letters are statistically different (p < 0.05). **(D)** Representative image of late EPC colony in Matrigel culture of mouse bone marrow lin^−^c-kit^+^ cells for 8 weeks and changes in late EPC colonies in cultured bone marrow lin^−^c-kit^+^ cells from wild-type and Sca-1 KO mice 24 h following i.v. challenge with heat-inactivated *E coli* (~1 × 10^8^ CFUs/mouse). N = 3. Data are mean ± SEM. Bars with different letters are statistically different (p < 0.05). **(E)** Increase in fluid retention in the lungs of Sca-1 KO mice 72 h following systemic *E coli* infection (i.v. ~1 × 10^8^ CFUs/mouse). N = 4. Data are mean ± SEM. *p < 0.05 vs. the wild-type group.

Sca-1 (or lymphocyte antigen 6 family member A, Ly6A.2) is a glycosylphosphatidylinisotol (GPI)-anchored cell membrane protein. Its natural ligand(s) remains unclear. Due to lack of an intracellular signaling domain, Sca-1 most likely acts as a co-regulator of lipid raft signaling. We performed co-immunoprecipitation (Co-IP) to determine the Sca-1 pulldown protein fractions in bone marrow cell lysate samples in mice 24 h following i.v. challenge with *E. coli*. The results revealed a unique 21-kDa Sca-1 pulldown protein fraction in wild-type mice (Sca-1^+/+^), which did not exist in Sca-1 KO (Sca-1^−/−^) mice (**Figure 9A**). Further analysis of polypeptides recovered from this Sca-1 pulldown fraction with proteomic mass spectrometry characterized that this pulldown fraction was enriched with Rac2. Rac2 belongs to the Rac subset of Rho small GTPase family, which acts as a molecular switch cycling between inactive, GDP-bound, and active, GTP-bound states. Activation of Rac is typically induced by guanine nucleotide exchange factors (GEFs) that are activated by receptor-dependent kinases. Activated Rac2 interacts with its target molecules to trigger cell responses via multiple signal pathways, including the ERK1/2 and JNK1/2 cascades (33, 34). GTPase-activating proteins (GAPs) stimulate hydrolysis of bound GTP to GDP, switching the Rac GTPase back to the inactive state. Particularly, GDP-dissociation inhibitors (GDIs) sequester the inactive Rac2 in the cytosol to prevent its activation and intracellular trafficking. Studies have shown that Rac2 translocation to the cell membrane is critical for the activation of ERK1/2 signaling (33). Immunohistochemistry identified that *E. coli* infection-induced upregulation of Sca-1 expression on the surface of marrow precursor cells tightly colocalized with cell membrane-recruited Rac2 in wild-type mice (**Figure 9B**). In Sca-1 KO mice, Rac2 was generally sequestered in the cytosol of cells following septic infection. These observations indicate that Sca-1 couples with Rac2 activation in signaling the EPC response. Cell surface crosslinking has been shown to promote Sca-1 signaling activity (25). The results of *in vitro* experiment via culturing marrow lin^−^c-kit^+^ cells with or without LPS plus Sca-1 crosslinking antibodies for 24 h exhibited that Sca-1 crosslinking significantly enhanced LPS-induced upregulation of VEGFR2 mRNA expression by these marrow SPCs (**Figure 9C**). Intravenous challenge with heated-inactivated *E. coli* significantly increased the activity of forming late EPC colonies in marrow lin^−^c-kit^+^ SPCs (**Figure 9D**). This increase in angiogenic/vasculogenic activity was inhibited in Sca-1 KO mice. Furthermore, Sca-1 KO mice showed an increase in fluid retention in the lungs compared to wild-type mice 72 h following systemic *E. coli* infection (**Figure 9E**).

## Discussion

Investigation on the pattern of marrow EPC response and the underlying signaling regulation has been facing many challenges. EPCs are rare cells. Peripheral blood samples from adult humans contain EPCs at 0.41 ± 0.47 CFUs/4 ml of blood as analyzed by cell culture (35) and 2.70 ± 0.73 CD34^+^VEGFR2^+^ cells/10^4^ PBMCs as determined using flow cytometry (36). In mice, similarly, EPCs are subtype cells in the LKS (enriched with hematopoietic stem cells) cell population. The entire LKS cell population only constitutes 0.08% of nucleated cells in the bone marrow (37). Furthermore, the population of these vascular precursors is considerably heterogeneous varying in their developmental stages and functional stats (10, 11, 38–40). Although efforts have been devoted to characterizing specific markers for EPCs, most of these strategies remain imperfect. Particularly, the commonly accepted signature markers for EPCs in humans are different from those in experimental animals. In addition, the availability of tissue samples, especially the bone marrow, from patients with septic infection remains strictly restricted or generally unacceptable. To characterize the dynamic pattern of the marrow EPC response, we employed a mouse model of systemic *E. coli* infection and observed that the marrow pool of EPCs with surface markers of LKS VGFR2^+^ and LKS CD133^+^VGFR2^+^ expanded rapidly in the early stage of septic infection (**Figures 1**, **2**). The significant increase in marrow EPCs occurred at 12 and 24 h post i.v. challenge with *E. coli*. By 48 h post septic infection, the marrow pool of EPCs returned to the control level. This sensitive response in rapidly generating marrow EPCs indicates the emergent requirement for EPCs by the host when facing septic challenge. With the marked increase in marrow EPCs, the release of these vascular precursors into the systemic circulation increased. The level of EPCs in the blood steam was significantly increased following septic infection in mice (**Figure 8A**). Our results in a mouse model of septic infection agree with clinical observations that increase in circulating EPCs occurs rapidly (6–12 h) following the diagnosis of sepsis (14, 16).

In the rapidly expanding marrow pool of EPCs following septic infection, cell proliferative activation, phenotypic convention, and enhancement of angiogenic/vasculogenic activity represent three prominent features. In this study, both systemic *E. coli* infection *in vivo* and exposure to LPS *in vitro* activated proliferation in EPC cell types as determined by their BrdU incorporation (**Figures 4A**, **6**). Proliferative activation evidently plays a fundamental role in the net increase in EPCs in the bone marrow. Our previous studies have shown that the TLR4-ERK1/2-cyclin D1 pathway mediates proliferative activation of marrow SPCs in response to septic infection (25–27). In this study, cyclin D1 expression by bone marrow lin^−^c-kit^+^ cells was significantly upregulated 24 h following i.v. challenge with *E. coli* in mice (**Figures 4B, C**). Previously, we have shown that enhancement of Sca-1 expression is accompanied by proliferative activation of SPCs in response to septic infection (25–27, 29). As mentioned earlier, EPCs are a subset of cells expressing VEGFR2 in the LKS cell population in the mouse bone marrow. Interestingly, the level of Sca-1 expression by EPCs (with surface markers of LKS VGFR2^+^ and LKS CD133^+^VGFR2^+^) was much higher than that by general LKS cell population (**Figure 3**). This uniqueness in higher level of Sca-1 expression appears critical for facilitating the signal regulation of EPC activity and the EPC response. The rapid increase in marrow EPC subset occurred at 12 and 24 h post septic infection (**Figure 2**), which was not parallel with the marked expansion of the total LKS population. Although the substantial increase in marrow LKS pool also occurred at 12 h, it continued 72 h following septic infection in our murine model. The early rapid increase in marrow EPC subset occurred concomitantly with the marked upregulation of Sca-1 expression by cells in the marrow LKS cell pool during the early stage of septic infection. *In vitro* culture of marrow SPCs with LPS plus recombinant murine VEGF confirmed that LPS exposure substantially upregulated Sca-1 expression by cultured cells (**Figure 5**). LPS plus VEGF stimulated proliferation in EPC type cells (increase in cell BrdU incorporation) in the culture system (**Figure 6**). Proliferating EPC-type cells expressed a much higher level of Sca-1 than their non-proliferating counterparts. The level of Sca-1 expression was positively correlated with proliferative activity in cultured marrow SPCs. Our results presented in **Figures 2**, **3** also exhibit a general tendency of stronger marrow EPC and SPC responses as well as the upregulation of Sca-1 expression by these cell types to the high dose of *E. coli* challenge, which suggests that the intensity of marrow EPC/SPC response may be dependent on the severity of the pathological challenge within a certain extent. Previously, we have reported that LPS exposure causes an LPS dose-dependent upregulation of Sca-1 expression by marrow SPCs (25). At present, information about the dynamic change in the bone marrow EPC response in patients with bacterial infection remains scant. However, early increase in the level of circulating EPCs has been repeatedly reported in patients with serious bacterial infections (12–17) as described previously.

In addition to the increase in BrdU^+^ EPCs, the number of BrdU^−^ EPCs also substantially increased in the bone marrow at the early stage of septic infection (**Figure 4A**). These BrdU^−^ EPCs were not generated from proliferation of existing EPCs. Instead, they were derived from phenotypic conversion of other cell types, most probably from the downstream lin^−^c-kit^+^Sca-1^-^ cells, via re-expression of Sca-1 and therefore reentry into the LKS cell pool. Indeed, exposure of marrow lin^−^c-kit^+^Sca-1^−^ cells to LPS markedly increased Sca-1 expression by these original Sca-1^−^ cells and produced a significant amount of EPC-type cells in the culture system (**Figures 5**, **6**). Expressing a high level of Sca-1 by phenotypically converted EPC-type cells also promoted proliferative activation of these cells as reflected by their presence with BrdU incorporation in the culture system. Our observations suggest that the phenotypic conversion at the SPC level serves as a powerful mechanism for urgently organizing the host defense response through efficiently using available SPC sources in mice. Although SPC phenotypic conversion in host defense response has not yet been directly described in humans, the phenomenon that the bone marrow switches its hematopoietic activity to polarize lineage development in various pathological conditions, such as granulopoietic activation with inhibition of erythropoiesis during bacterial infection and erythropoietic activation with relative inhibition of cell development in other lineages during anemia caused by loss of blood, has been well known for many decades. Further investigation along this line will provide a deep insight into the molecular signaling mechanisms underlying marrow SPC reprograming in humans during the host defense response.

During the EPC response to septic infection, angiogenic/vasculogenic activity in marrow EPCs was significantly enhanced in mice. Marrow lin^−^c-kit^+^ cells from donor mice challenged with heat-inactivated *E. coli* for 24 h exhibited a stronger vasculogenic activity in Matrigel plugs that were implanted subcutaneously for 5 weeks in the recipient mice (**Figure 4**). *In vitro* culture of these precursors in Matrigel media also demonstrated a significant increase in the activity of forming late EPC colonies (**Figure 9D**). VEGF is a potent mediator for angiogenic/vasculogenic activation of vascular precursors. In the early stage of septic infection, VEGF level in the systemic circulation was substantially elevated (**Figure 7**). In the meantime, the number of VGFR2-expressing cells in marrow lin^−^c-kit^+^ SPC pool increased substantially (**Figures 2C, D**). Exposure of marrow lin^−^c-kit^+^ SPCs to LPS plus VEGF for 24 h significantly increased VEGFR-expressing cells in the culture system (**Figures 6A–C**). Obviously, the polarized change in tissue generation of VEGF and cell expression of VEGFR2 during the early stage of host defense response provides an optimal environment for promoting angiogenic/vasculogenic activation of marrow EPCs.

With the rapid expansion of marrow EPC subpopulations, release of EPCs into the systemic circulation significantly increased following septic infection. To determine the significance of this increase in release of EPC into the circulation, we monitored the dynamic change in recruitment/homing of marrow-derived cells in the lungs following septic infection in the GFP^+^ marrow chimeric mouse model. Massive numbers of marrow-derived cells were recruited into the lungs during the acute inflammatory phase of 24 to 72 h (**Figure 8**). Even in this early stage of host response, many pulmonary-recruited marrow cells appeared to merge in (colocalization with) the capillary wall. In the late stage of 7 to 14 days post septic infection, a large amount of marrow-derived angiogenic cells clearly integrated into the microvasculature in lung tissue. These results confirm that marrow-derived angiogenic cells actively participate in maintaining the integrity of microvasculature via structural integration. The protective effort provided by marrow EPC response evidently occurs early as a component of host defense mechanism in addition to repairing established vascular damage in the late “repairment stage.” In supporting our investigation, previous studies on chimeric mice with reconstituted bone marrow cells expressing GFP have reported that bone marrow-derived cells start appearing in the lungs at 24 h following intranasal insufflation of LPS (41). Numerous marrow-derived cells are integrated in lung parenchyma 1 week thereafter. Immunohistological analysis indicates that these recruited marrow precursors differentiate toward capillary endothelial cells. Investigations have also shown that EPCs promote angiogenic activity and repairing vascular injury via generating soluble mediators and extracellular vesicles to exert paracrine as well as autocrine effects (10, 11). EPC homing as well as retaining in the tissue site of infection and inflammation will facilitate local release of angiogenic factors by these vascular precursors to fulfill their paracrine as well as autocrine effects. In addition to producing angiogenic factors, EPCs have been reported to exert immunomodulatory effects via producing anti-inflammatory cytokines, including IL-4 and IL-10 (42). With secretion of these anti-inflammatory cytokines, EPCs interplay with immune cell types via promoting differentiation of macrophages toward the M2 phenotype and proliferation of T-regulatory cells (Tregs). The effort of these anti-inflammatory actions may potentially contribute to mitigating tissue injury.

Following septic infection, plasma level of G-CSF was markedly increased (**Figure 7**). The plasma level of SDF-1 also increased at 24 h post i.v. *E. coli* challenge. G-CSF has been known as an active mediator promoting mobilization of EPCs into the systemic circulation (43, 44). Increase in SDF-1 in circulation has also been reported to facilitate release of EPCs from the bone marrow (45, 46). In lung tissue, VEGF level only slightly increased (with statistical significance) at 6 h following septic infection, which suggests that the enhancement of EPC angiogenic/vasculogenic activity achieved prior to their homing to the lungs is critical for fulfilling defense function of these vascular precursors in the pulmonary microvasculature. Septic infection caused a marked increase in G-CSF level in lung tissue. It remains to be defined if this increase in G-CSF level exerts any effect on recruited EPCs in pulmonary microvasculature. No alteration in SDF-1 level in the lungs was detected during 48 h of septic infection, which suggests that it might not exert any significant influence on recruiting EPCs in pulmonary microvasculature in the early course of host defense response.

One unique feature of the marrow EPC response to septic infection was the strong expression of Sca-1 by EPC cell types. This markedly enhanced Sca-1 expression was correlated with proliferative activation, phenotypic conversion, and angiogenic/vasculogenic reprogramming of EPC cell types in the marrow SPC pool during the host defense response. Co-IP, mass spectrometry, and immunohistology analyses identified the tight coupling of Sca-1 with Rac2 in its cell surface translocation and, therefore, securing the active status of Rac2 (**Figures 9A, B**). Activation of Rac2 has been known as a turn-on key for the activation of multiple signal pathways, including the ERK1/2 and JNK1/2 cascades (33). Previous studies from our group have demonstrated that the ERK1/2 and JNK1/2 cascades provide strong signaling for SPC proliferative activation and phenotypic conversion (25, 26, 29). Sca-1 KO abolished the coupling of Sca-1 with Rac2 in the process of Rac2 cell surface translocation for activation (**Figures 9A, B**). Septic challenge-induced activation of late EPC colony-forming activity in marrow lin^−^c-kit^+^ SPCs was impaired (**Figure 9D**). In addition, Sca-1 KO mice showed an increase in fluid retention in the lungs in comparison to wild-type mice 72 h following septic infection (**Figure 9E**). Our current study on the marrow EPC response was conducted in the murine model of septic infection. However, human cells express GPI-anchored Ly6/uPAR family proteins including CD59, CD52, and LY6A (47–50). These molecules have been documented to congregate on lipid rafts in the cell surface promoting their interactions with other proteins for cell signaling (47). Further investigation on functions of GPI-anchored Ly6/uPAR family proteins in human marrow SPCs will facilitate understanding of the molecular signaling mechanisms underlying the EPC response in patients with septic infection/sepsis.

## Data availability statement

The data presented in the study are deposited in the Dryad Digital Repository. Accession number (link) is https://doi.org/doi:10.5061/dryad.m63xsj49c.

## Ethics statement

The animal study was approved by Institutional Animal Care and Use Committee of Northeast Ohio Medical University, Institutional Animal Care and Use Committee of Michigan State University, and Institutional Animal Care and Use Committee of Louisiana State University Health Sciences Center. The study was conducted in accordance with the local legislation and institutional requirements.

## Author contributions

XS: Conceptualization, Data curation, Formal analysis, Funding acquisition, Investigation, Methodology, Resources, Supervision, Validation, Writing – original draft, Writing – review & editing. KS: Data curation, Formal analysis, Investigation, Validation, Visualization, Writing – review & editing. TE: Data curation, Formal analysis, Investigation, Methodology, Validation, Visualization, Writing – review & editing. Y-PL: Data curation, Formal analysis, Investigation, Methodology, Validation, Visualization, Writing – review & editing. Y-LC: Data curation, Formal analysis, Investigation, Methodology, Validation, Visualization, Writing – review & editing. JM: Data curation, Investigation, Methodology, Validation, Visualization, Writing – review & editing. RS: Data curation, Formal analysis, Investigation, Methodology, Validation, Writing – review & editing. PZ: Conceptualization, Data curation, Formal analysis, Funding acquisition, Investigation, Methodology, Project administration, Resources, Supervision, Validation, Visualization, Writing – original draft, Writing – review & editing.
